# The NOTCH1-HEY1 pathway regulates self-renewal and epithelial-mesenchymal transition of salivary adenoid cystic carcinoma cells

**DOI:** 10.7150/ijbs.36407

**Published:** 2020-01-01

**Authors:** Jing Xie, Li-song Lin, Xiao-yu Huang, Rui-huan Gan, Lin-can Ding, Bo-hua Su, Yong Zhao, You-guang Lu, Da-li Zheng

**Affiliations:** 1 Department of Preventive Dentistry, School and Hospital of Stomatology, Fujian Medical University, 246 Yang Qiao Middle Road, Fuzhou 350000, China; 2 Department of Oral and Maxillofacial Surgery, Affiliated First Hospital of Fujian Medical University, 20 Cha Zhong Road, Fuzhou 350005, China; 3 Key laboratory of Stomatology of Fujian Province, School and Hospital of Stomatology, Fujian Medical University, 88 Jiaotong Rd, Fuzhou 350004, China; 4 Key Laboratory of Ministry of Education for Gastrointestinal Cancer, Fujian Medical University, 1 Xue Yuan Road, University Town, Fuzhou 350122, China; 5 Department of pathology, School and Hospital of Stomatology, Fujian Medical University, 246 Yang Qiao Middle Road, Fuzhou 350000, China

**Keywords:** SACC, NOTCH1, HEY1, cancer stem cells, proliferation, invasion, EMT

## Abstract

Our previous study demonstrated a close relationship between the NOTCH signaling pathway and salivary adenoid cystic carcinoma (SACC). Its receptor gene, NOTCH1, and its downstream gene, HES1, contribute to the proliferation, invasion and metastasis of SACC. Accumulating evidence supports HEY1 as another effector of the signaling pathway. The purpose of this study was to explore the effects of the NOTCH1-HEY1 pathway on the proliferation, invasion and metastasis of SACC cells. Our results verified that HEY1 is a specific molecular target of the NOTCH signaling pathway in SACC cells and that its expression in carcinoma is much higher than that in paracarcinoma tissues. The expression of NOTCH1 and HEY1 are positively correlated in the salivary adenoid cystic carcinoma tissues. NOTCH1 is significantly related to the activation of HEY1 in SACC, and that HEY1 reciprocally regulates NOTCH1 expression in SACC. HEY1 promotes cell proliferation and spheroid formation and inhibits cell apoptosis *in vitro*. In addition, HEY1 enhances the tumorigenicity of SACC *in vivo*. Furthermore, HEY1 increases cell invasion and metastasis by driving the expression of epithelial-mesenchymal transition (EMT)-related genes and MMPs. The results of this study indicate that the NOTCH1-HEY1 pathway is specifically upregulated in SACC and promotes cell proliferation, self-renewal, invasion, metastasis and the expression of EMT-related genes and MMPs. Our findings suggest that a NOTCH1-HEY1 pathway inhibitor might therefore have potential therapeutic applications in treating SACC patients by inhibiting cancer cell growth and metastasis.

## Introduction

SACC is an aggressive tumor with unpredictable and unique biological destruction that exhibits features such as slow but relentless growth, a propensity for peripheral nerve and blood vessel invasion, a high incidence of distant lung metastasis and a high risk of relapse 1-4. As a result, the survival rates after a diagnosis of SACC are 35% at 5 years, 15% at 10 years, and 0% at 15 years, which points to the poor outcomes of the tumor 3. Due to the poor understanding of the mechanism of SACC progression, no effective therapy has been provided 5. Therefore, it is impending to define the underlying mechanism of SACC and to explore the methods for SACC treatment.

NOTCH signaling critically influences cellular differentiation and proliferation, embryonic development and blood vessel formation 6. Disordered NOTCH signaling affects the occurrence, development, invasion and metastasis of many different types of tumors, as well as inducing tumors directly or indirectly via interactions with other signaling pathways 7-9. Multiple factors of NOTCH signaling, including ligands, receptors, signal transducers and effectors execute pleiotropic effects 10. In our previous study, we explored the role of NOTCH1 11 and its primary effector HES1 in SACC. HEY is normally considered another primary target of NOTCH signaling as well as a related but distinct member of the bHLH family compared with HES, which can also play a role as a transcriptional repressor in cell fate decisions 9, 10, 12, 13. The HEY family consists of HEY1, HEY2 and HEYL, which are encoded by three distinct genes with similar structures 14 and encode a nuclear protein belonging to the hairy and enhancer of split-related (*HESR*) family of basic helix-loop-helix (bHLH)-type transcriptional repressors.

Although increasing advances toward the function of HEY1 have been reported in electronic public literature searches, the mechanism of HEY1 in SACC remains uncovered. Our group used a siRNA technique to inhibit the expression of HEY1 in SACC cells on account of the findings of RNA-Seq. Through a series of experiments *in vitro* and *in vivo*, we aimed to illuminate the functional role of the NOTCH1-HEY1 pathway in salivary adenoid cystic carcinoma, which will open a new avenue to our understanding of the NOTCH signaling pathway in SACC.

## Materials and Methods

### Cell culture and clinical samples

The SACC-LM cell line was obtained from the Peking University Health Science Center. The cells were maintained in RPMI-1640 medium (Gibco BRL, Grand Island, NY) supplemented with 10% fetal bovine serum (Gibco) and incubated in a humidified atmosphere of 95% air and 5% CO_2_ at 37°C. Experiments were performed using cells in the exponential phase of growth. Tissue samples were obtained from Fuzhou PLA General Hospital and Fujian Medical University Union Hospital. Seventy-seven normal salivary tissues and eithty-seven SACC samples were included. This study was approved by the Institutional Review Board of Fujian Medical University, and written informed consent was obtained from each participant.

### RNAi transfection

The negative control (NC) siRNA and two siRNAs against HEY1 were synthesized (GenePharma, Shanghai, China). The siRNA sequences are listed in Table 1. Cells were transfected with siRNAs using Lipofectamine RNAiMAX (Invitrogen, USA) according to the manufacturer's instructions.

### Co-transfection of plasmid DNA and siRNA

The pcDNA3.1-NICD1 plasmid was a kind gift from Dr. Glenn Doughty, Harvard Medical School 15. The co-transfection combinations are the vector plasmid and siRNA NC (named: VE NC), the vector plasmid and siRNA targeted HEY1 (named: VE siRNA-HEY1), the vector plasmid and siRNA targeted HES1 (named: VE siRNA-HES1), the pcDNA3.1-NICD1 plasmid and siRNA NC (named: N1 NC), the pcDNA3.1-NICD1 plasmid and siRNA targeted HEY1 (named: N1 siRNA-HEY1) and the pcDNA3.1-NICD1 plasmid and siRNA targeted HES1 (named: N1 siRNA-HES1). Cells were transfected with plasmid DNA and siRNAs at the same time using Lipofectamine 2000 (Invitrogen, USA) according to the manufacturer's instructions.

### Inhibitor of Mastermind Recruitment-1 (IMR-1) treatment

The inhibitor of Mastermind Recruitment-1 (IMR-1) purchased from MedChemExpress (New Jersey, USA) was dissolved in DMSO (dimethyl sulfoxide) at a concentration of 10mM, and stored at -20 °C. For *in vitro* experiment, seeded cells to be 30-40% confluent, the cells were treated with 10 μM and 20 μM IMR-1 at indicated time. The DMSO as a vehicle was used as a negative control.

### Immunohistochemistry

For the immunohistochemical assays, 5-μm-thick tissue sections were mounted onto slides coated with poly-L-lysine. After deparaffinization in xylene, the sections were rehydrated in a decreasing gradient of ethanol and washed for 10 min in phosphate-buffered saline (PBS) (pH 7.2). Endogenous peroxidase activity was inhibited by incubation in methanol containing 3% H_2_O_2_ for 10 min. After several washes in PBS, the sections were blocked with a universal blocking reagent (Maxin, USA) for 10 min at room temperature and then incubated with primary antibodies against Caspase-3 (1:300, Cell Signaling, USA), Caspase-9 (1:500 dilution, Abcam, UK), Ki-67 (1:500 dilution, Abcam, UK), NOTCH1 (1:400 dilution, Sigma, USA) and HEY1 (1:30 dilution , Abcam, UK) for 1 h at room temperature. After several washes in PBS, the sections were incubated with a biotin-conjugated secondary antibody (Maxin) for 10 min at room temperature. After several washes in PBS, the sections were incubated with streptavidin-peroxidase (Maxin) for 10 min at room temperature. The sections were rinsed with PBS, and the antibody complexes were visualized by incubation with diaminobenzidine tetrahydrochloride (DAB) chromogen (Maxin). The sections were then counterstained with hematoxylin (Dako, Denmark), dehydrated, and examined by light microscopy. All slides were reviewed independently by two pathologists who were blinded to each other's readings. The staining results were assessed on a three-tier scale: negative indicated no staining, 1+ indicated weak staining and 2+ indicated strong staining. Immunohistochemical results were graded with 3 different scores (negative, positive and strong positive) as follows: negative indicated no staining or 1+ staining in ≤30% of cells, positive indicated 1+ staining in >30% of cells or 2+ staining in <50% of cells and strong positive indicated 2+ staining in >50% of cells.

### Quantitative real-time PCR analysis

Total RNA was extracted from cells with Trizol reagent (Invitrogen, USA) and reverse transcribed into cDNA with the PrimeScript RT reagent kit (TaKaRa, Japan). The cDNA was used as the template to detect the expression of the genes of interest by qRT-PCR with SYBR Premix Ex Taq™ (TaKaRa, Japan). The primers used in this study are listed in Table 2. Data were analyzed according to the 2^-△△Ct^ method.

### Western blot assay

Total protein was separated by 8% SDS-PAGE and transferred onto PVDF membranes (Amersham, USA). Subsequently, the membranes were immunoblotted with primary antibodies against E-Cadherin (1:1000 dilution, Cell Signaling, USA), N-Cadherin (1:1000 dilution, Cell Signaling, USA), Twist1 (1:1000 dilution, Cell Signaling, USA), NOTCH1 (1:1000 dilution, Sigma, USA), HES1(1:1000 dilution, Abcam, UK) and HEY1 (1:250 dilution, Abcam, UK) and β-actin (1:2000 dilution, Sigma, USA) in 5% bovine serum albumin overnight, washed three times with Tris-buffered saline containing 0.1% Tween 20, and incubated with the secondary antibody (1:2000 dilution, Boster, China). The immunoreactive protein bands were visualized using CDP STAR reagent (Roche, IN, USA), and signals were scanned with a densitometer for the semiquantification of signal intensity.

### Cell viability assay

Cell proliferation was measured by counting viable cells with a Cell Counting Kit-8 (Dojindo, Kumamoto, Japan). Cells were first transfected with the indicated siRNA for 24 h and then planted into a 96-well plate. In the experiment of small molecule inhibitors for the treatment of SACC cell, the cells were seeded into a 96-well plate for 16 h, then treated with different doses of inhibitor IMR-1. At the same time each day for 5 consecutive days, the original culture medium was removed, and 10 μl of CCK8 and 90 μl of fresh RPMI-1640 medium were added to each well. The cells were incubated at 37°C for 1 h. The absorbance of each well was measured with a microplate reader (Pharmacia Biotech, USA) at 450 nm.

### Colony formation assay

Twenty-four hours after siRNA transfection, the cells were plated into 6-cm plates (600 cells per plate) and cultured for 2 weeks. In the experiment of small molecule inhibitors for the treatment of SACC cell, the cells were seeded into a 6-well plate for 16 h, then treated with different doses of inhibitor IMR-1 and cultured for 2 weeks. Colonies were fixed with cold methanol for 10 min and stained with 1% crystal violet for 30 min.

### *In vitro* cell invasion assay

Cell invasion was determined using 24-well Matrigel-coated transwell chambers (8-μm pore size, BD Science, USA). Twenty-four hours after siRNA transfection, cells were serum starved for 24 h and then collected in RPMI-1640 medium containing 1% FBS. Cells were plated in the upper chamber at a density of 1.0×10^5^ cells per well, and 800 μl of RPMI-1640 medium containing 10% FBS was added to the lower chamber. After incubation at 37°C for 48 h, the Matrigel and cells in the upper chamber were removed using a cotton swab and stained with 1% crystal violet for 10 min. Cells were counted and photographed by microscopy in at least five random fields (×200).

### *In vitro* cell migration assay

Cell migration assays were performed using 24-well transwell chambers (8-μm pore size, BD Science, USA). The procedure used for this assay was similar to that of the cell invasion assay, except the transwell was not coated with Matrigel.

### Cell apoptosis assay

Cellular apoptosis was analyzed using a FITC Annexin V Apoptosis Detection Kit (BD Pharmingen™, USA). At 48 h posttransfection, the cells were collected and washed in PBS and then stained with annexin V and propidium iodide for 15 min. The percentage of apoptotic cells was quantified using a BD FACS Verse flow cytometer.

### 3D Spheroid formation assay

A tumor spheroid formation assay was performed using ultra-low attachment (ULA) 6-well round-bottomed plates (Corning, USA). Twenty-four hours after siRNA transfection, the cells were seeded at a density of 40000 cells per well in 4 ml of DMEM/F12 without FBS in a ULA plate. The 3D tumor spheroids were incubated for 14 days before analysis.

### Establishment of a xenograft tumor model

Female BALB/c nude mice 6-8 weeks of age were purchased from the Center for Animal Experiments of Fujian Medical University. Cells (2×10^6^) were suspended in 0.2 ml of serum-free DMEM and injected into the right axillary fossa of each mouse. Tumor size was calculated using the formula V =width^2^× length/2. At the end of the experiment, the tumors were harvested and weighed. The experimental animal protocols were approved by the Animal Care and Use Committee of Fujian Medical University.

### Statistical analysis

The statistical analysis of HEY1 immunoreactivity was performed using the rank-sum test. The statistical analysis of PCR results and the *in vitro* cell migration/invasion assays was determined by One-Way ANOVA followed by Tukey's multiple Comparison test. p<0.05 was considered statistically significant and is indicated in the figures as ns when P>0.05, * when P<0.05, ** when P<0.01 and *** when P<0.001.

## Results

### The expression of HEY1 is regulated by NOTCH1 in SACC cells

We previously reported that NOTCH1 and its downstream gene HES1 both act as an oncogene in SACC and accelerate the proliferation and migration of SACC cells 16. We performed and analyzed the RNA-Seq results pertaining to NOTCH1 upregulation in SACC cells, and another NOTCH1 downstream gene, HEY1, drew our attention. We validated satisfactory RNA-Seq results and deposited the data in the NCBI Sequence Read Archive (SRA, https://www.ncbi.nlm.nih.gov/sra) under accession no. SRR5572289, which revealed a total of 1323 coding genes that displayed differential expression in NOTCH1-overexpressing cells. In addition, Gene Ontology (GO) analysis provided a description of cell function: the highly expressed genes are involved in cell mobility, cell differentiation, proliferation and signal transduction, while the weakly expressed genes are involved in metabolism, transport and differentiation.

**Figure 1:**
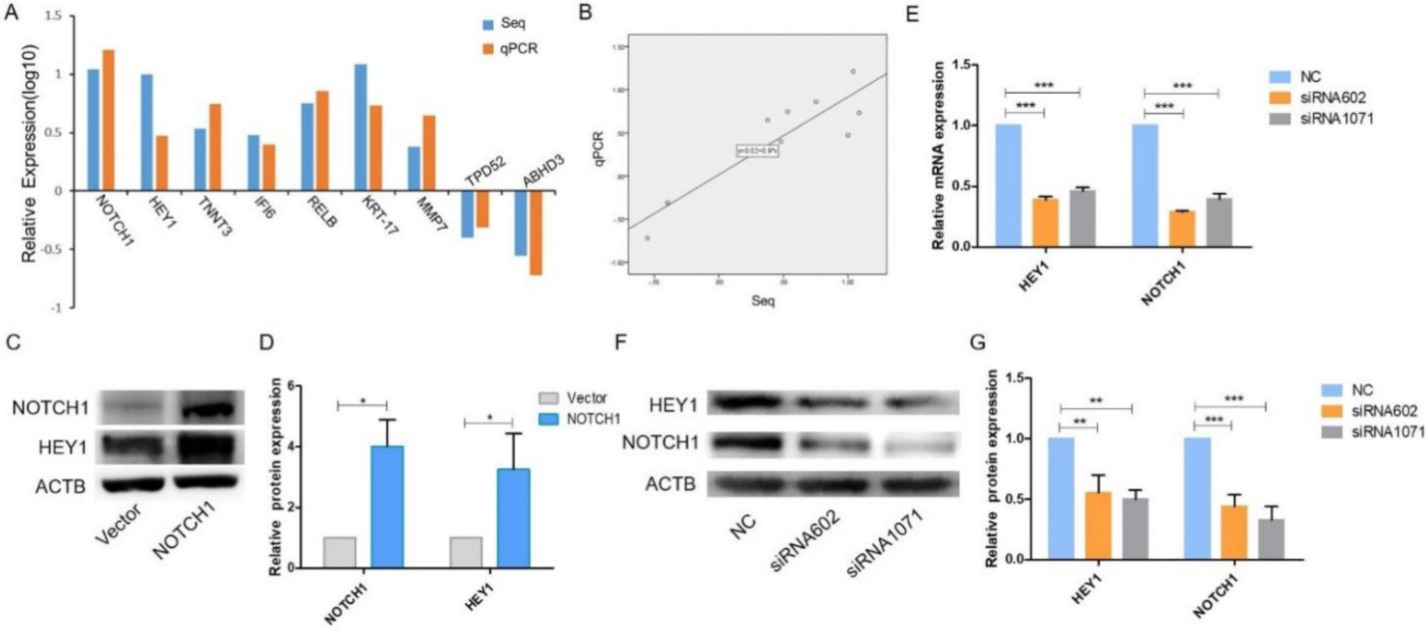
** The NOTCH1-HEY1 pathway is a positive feedback loop in SACC cells.** (A) The expression of NOTCH1, HEY1, TNNT3, IFI6, RELB, KRT-17, MMP7, TPD52 and ABHD3 genes, which were selected from the RNA-Seq results, was measured by qRT-PCR. (B) Linear regression analysis was used to compare the expression levels of the abovementioned genes between the RNA-Seq and qRT-PCR results. (C, D) Western blot analysis and the quantification of NOTCH1 and HEY1 expression in SACC cells after NOTCH1 upregulation by pcDNA3.1-NICD1 plasmid transfection. (E) The mRNA expression of NOTCH1 and HEY1 after HEY1 downregulation by siRNAs was detected by RT-PCR. (F, G) The protein expression of NOTCH1 and HEY1 when HEY1 inhibition was measured by western blot analysis. *P < 0.05, ** P < 0.01, and *** P < 0.005.

On account of the RNA-Seq results, another 8 genes were randomly selected along with NOTCH1, and qRT-PCR was performed to verify their expression. The expression levels of HEY1, TNNT3, IFI6, RELB, KRT-17, MMP7, TPD52 and ABHD3 obtained by qPCR were consistent with the RNA-Seq results (Fig. 1 A, P<0.001), which suggested that HEY1 is a specific downstream gene of the NOTCH1 signaling pathway. Linear regression analysis of the relationship between RNA-Seq and qPCR revealed a reliable RNA-Seq result (Fig. 1B, R^2^=0.805). Next, we employed western blot analysis to examine the expression of HEY1 in SACC cells (Fig. 1C) and found that the expression of HEY1 was significantly enhanced in the group in which NOTCH1 was overexpressed compared with the control group. In addition, as described in our previous qPCR results, HEY1 expression is downregulated when NOTCH1 is restrained in SACC cells 11. Taken together, these data suggest that NOTCH1 is significantly associated with the expression of HEY1.

### HEY1 inhibition decreases NOTCH1 expression in SACC cells

It has been reported that all NOTCH receptors were significantly associated with HEY1, and HEY1 could reciprocally regulate the expression of NOTCH4 in HNSCC 17. Therefore, we wondered whether HEY1 also has this molecular mechanism that reversely regulated NOTCH1 in SACC. We applied siRNA-mediated knockdown of HEY1 in SACC-LM cells in the following experiments. As shown by real-time RT-PCR (Fig. 1E) and western blot (Fig. 1F-G) analyses, siRNAs targeting HEY1 (siRNA-602 and siRNA-1071) efficiently reduced the expression of HEY1 in SACC-LM cells compared with the negative control (NC). In previous studies, it was reported that the expression of HEY1 was regulated by NOTCH1 receptors 18-19. However, we respectively determined the mRNA and protein levels of NOTCH1 in SACC-LM cells after HEY1 was suppressed by siRNAs through real-time PCR (Fig. 1E) and western blotting (Fig. 1F-G). The results showed that the expression of NOTCH1 was obviously reduced. Thus, these results indicate that HEY1 may reverse regulate NOTCH1 in SACC cells, unlikely previous reports. Therefore, we focused on the effector of NOTCH signaling, HEY1, for a series of further studies.

### NOTCH1-HEY1 is upregulated in human adenoid cystic carcinoma tissues

**Figure 2:**
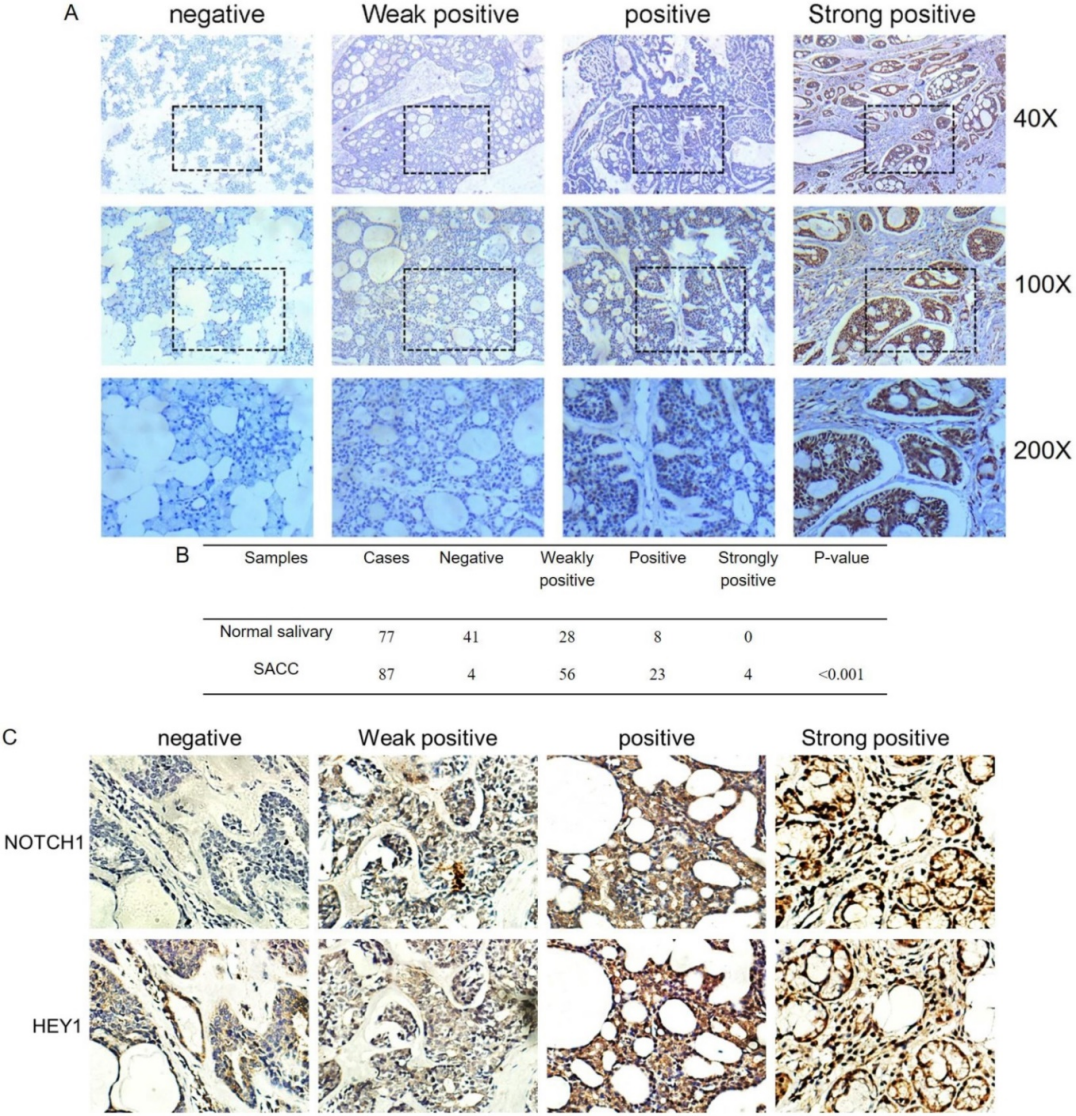
** HEY1 is upregulated in adenoid cystic carcinoma tissues and is positively correlated with NOTCH1.** (A) Representative images of HEY1 expression, as determined by immunohistochemistry and the following four-tier scale: negative, weakly positive, positive and strongly positive. (B) The statistics of the expression of HEY1 in adenoid cystic carcinoma tissues and normal tissues. (C) Representative images of NOTCH1 and HEY1 expression, as determined by immunohistochemistry and the following four-tier scale: negative, weakly positive, positive and strongly positive.

The immunohistochemistry results of NOTCH1 and HES1 in our previous study demonstrated that higher expression levels were related to metastatic and recurrent SACC. As another pivotal member of the NOTCH1 signaling path, we investigated the expression of HEY1 in salivary adenoid cystic carcinoma, including 77 samples of normal tissue and 87 adenoid cystic carcinoma cases, via immunohistochemistry. According to the staining results, we classified the expression of HEY1 into a four-tier scale. As shown in Fig. 2A and 2B, HEY1 expression was absent or low in normal salivary gland tissues, while higher expression levels were observed in the adenoid cystic carcinoma cases (P<0.001). Then, we would like to further confirm whether there was a positive correlation between NOTCH1 and HEY1 in salivary adenoid cystic carcinoma tissues. Immunohistochemistry was used to detect the expression of NOTCH1 and HEY1 in 27 cases of the adenoid cystic carcinoma tissues. The results showed that the expression of NOTCH1 and HEY1 were positively correlated in the salivary adenoid cystic carcinoma tissues (Fig. 2C, P<0.01, n=27). In conclusion, these results indicated that the NOTCH1-HEY1 signaling pathway was specifically up-regulated in SACC.

### HEY1 regulates cellular apoptosis

**Figure 3:**
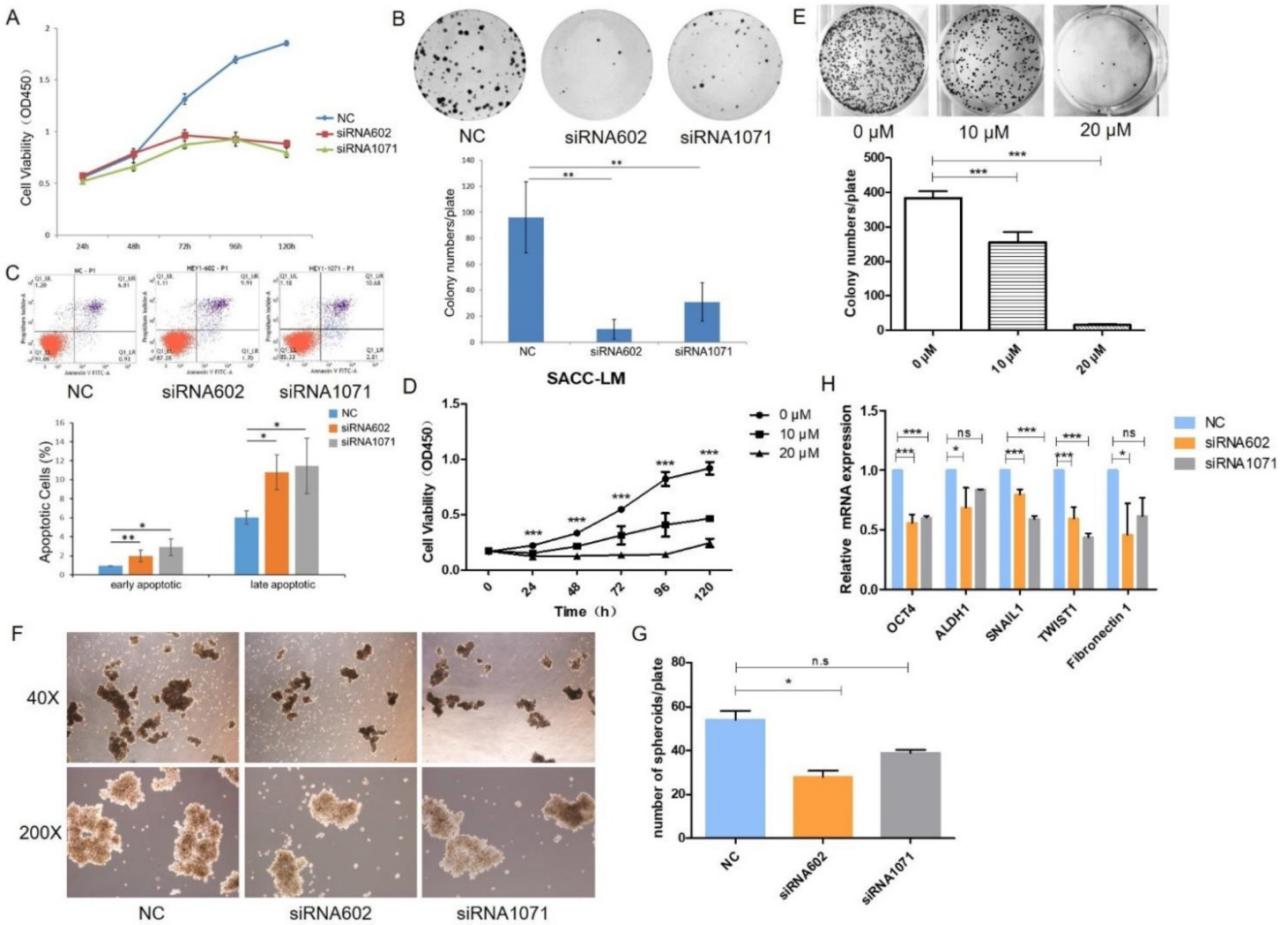
** HEY1 promotes cell proliferation, self-renewal and regulates cellular apoptosis, and IMR-1 inhibits cell growth.** After siRNA transfection, the cell proliferation was measured by CCK8 (A, P<0.001 by One-Way ANOVA followed by Tukey's multiple Comparison test from days 3, 4 and 5) and colony formation assays (B, P<0.01 by One-Way ANOVA followed by Tukey's multiple Comparison test, n=3). The percentages of early and late apoptosis cells were detected by flow cytometry (C, P<0.05 by One-Way ANOVA followed by Tukey's multiple Comparison test). After treated with IMR-1, the cell proliferation was measured by CCK8 (D, P<0.001 by One-Way ANOVA followed by Tukey's multiple Comparison test from days 2, 3, 4 and 5) and colony formation assays (E, P<0.01 by One-Way ANOVA followed by Tukey's multiple Comparison test, n=3). (F) Representative images of tumor spheroids of the NC, siRNA602 and siRNA1071 groups. The numbers of cell spheroids in the NC, siRNA602 and siRNA1071 groups (G, P<0.05 by One-Way ANOVA followed by Tukey's multiple Comparison test). (H) The mRNA expression of OCT4, ALDH1, Snail1, Twist1 and Fibronectin 1 when HEY1 was downregulated in SACC cells. *P < 0.05, ** P < 0.01, and *** P < 0.005.

To explore the functional effects of HEY1 on the proliferation of cancer cells, the cells were transfected with siRNAs, and the growth of SACC-LM cells was significantly inhibited in the siRNA602 and siRNA1071 groups according to the CCK8 assay (Fig. 3A, P<0.001 at days 3, 4 and 5). Additionally, the same results were detected in colony formation assays (Fig. 3B, P<0.01, n=3). Furthermore, we knocked down HEY1 by siRNA transfection for 48h and then quantified apoptotic cells using annexin V and PI staining and flow cytometric analysis. Compared with the negative control cells (Fig. 3C), after 48 h of transfection, the percentage of both early apoptosis (annexin V-positive and PI-negative) and late apoptosis (annexin V-positive and PI-positive) cells was increased in HEY1-silenced cells. These assays collectively support that HEY1 plays an oncogenic role in SACC.

### IMR-1 inhibits the proliferation of SACC cell *in vitro*

In the above experiments, we found that knockdown of HEY1 was effectively inhibited the proliferation of SACC cells. So, we wondered whether the inhibitory effect of knockdown of HEY1 on SACC cells was consistent with the NOTCH signaling inhibitor. Inhibitor of Mastermind Recruitment-1 (IMR-1) is a new class of NOTCH pathway inhibitor that target transcriptional activation 20. The results of CCK8 assay (Fig. 3D, P<0.001 at days 2, 3, 4, 5 and 6) and colony formation assay (Fig. 3E, P<0.01, n=3) showed that treatment of SACC cells with IMR-1 displayed a dose-dependent reduction. The NOTCH signaling pathway inhibitor IMR-1 can effectively inhibit the growth of SACC cells, which is consistent with the effect of HEY1-specific siRNAs on cell proliferation. Therefore, it is demonstrated from another perspective that HEY1 regulates the proliferation of SACC cells through the NOTCH signaling pathway.

### HEY1 knockdown inhibits SACC cell spheroid formation and growth *in vitro*

Some studies have shown that spheroids of cancer cells, because of their morphological and biological characteristics that are similar with those of solid tumors, are a promising model for the simulation of tumor changes *in vivo* 21. The results of the tumor cell spheroid formation assay showed that the volume (Fig. 3F) and number (Fig. 3G) of tumor spheroids were decreased in the inhibition group compared to the control group. Furthermore, we also detected the mRNA expression of several stemness-related genes, and the results showed that the knockdown of HEY1 in SACC-LM cells inhibited the expression of OCT4, ALDH1, Snail1, Twist1 and Fibronectin 1 (Fig. 3H). Taken together, these results suggest that the knockdown of HEY1 can effectively inhibit 3D tumor cell spheroid formation and stemness in SACC stem cells.

### Knockdown of HEY1 inhibits tumorigenicity *in vivo*

**Figure 4:**
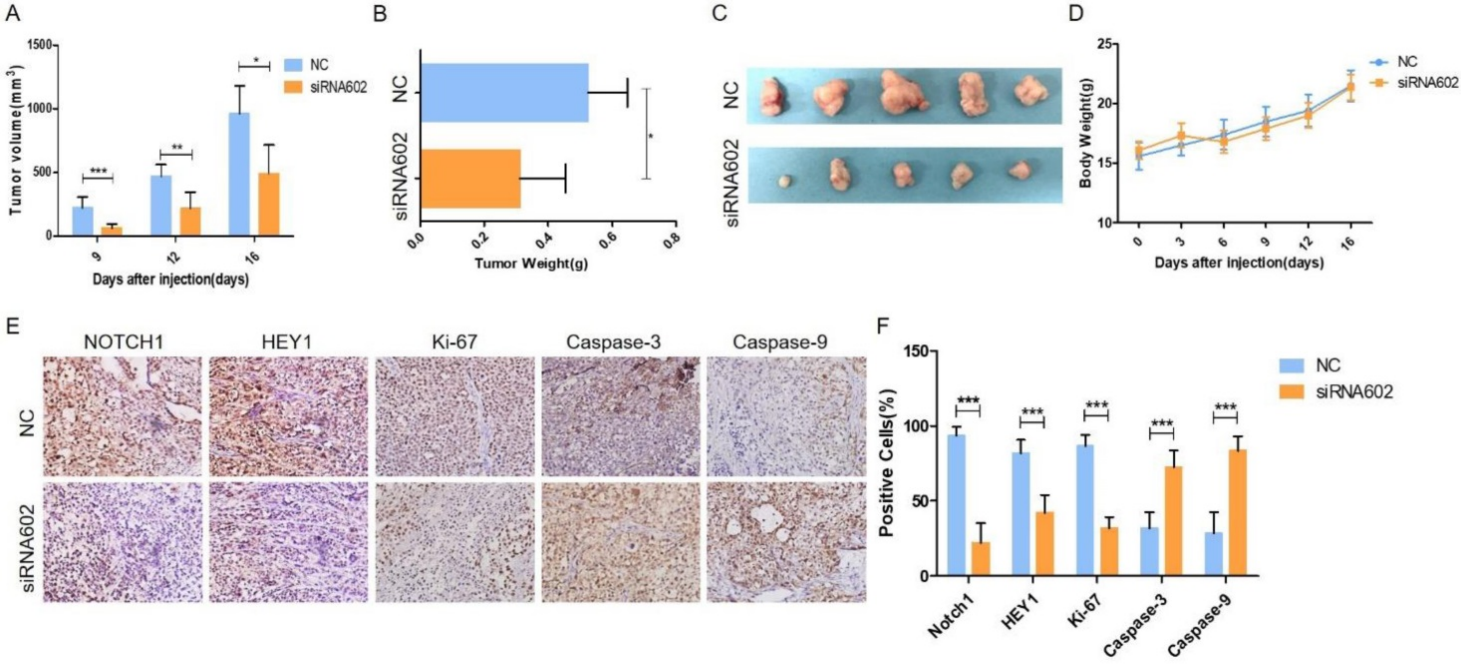
** Knockdown of HEY1 inhibits the growth of tumors *in vivo*.** (A) Changes in the tumor volume of nude mice were measured during the experiment. The nude mice were sacrificed, and the tumors were removed, weighed (B, P < 0.05 by t-test) and photographed (C). Changes in the body weights of nude mice in the NC and siRNA602 groups (D, P>0.05 at days 0, 3, 6, 9, 12 and 16 by t-test). (E) The expression of NOTCH1, HEY1, Ki-67, Caspase-3 and Caspase-9 was detected in the xenograft tumors by IHC (DAB, 200×), and the positive cells were counted and compared among the groups (F, P < 0.001 by t-test). *P < 0.05, ** P < 0.01, and *** P < 0.005.

*In vitro* cell studies demonstrated that downregulating the expression of HEY1 in SACC-LM cells can inhibit cell proliferation, promote cell apoptosis, block the cell cycle, and inhibit spheroid formation. To further study the oncogenic effect of HEY1 on tumorigenicity *in vivo*, SACC-LM cells were separately transfected with different siRNAs (NC and siRNA-602) and then subcutaneously injected into the flanks of nude mice. Throughout the experimental observation period, we weighed the nude mice every 3 days and measured the length and width of the subcutaneous tumor. As seen from the trend of tumor growth (Fig. 4A), the tumor growth rate of the siRNA602 group was significantly lower than that of the NC group, and the weight of nude mice was not obviously changed (Fig. 4D). Moreover, the wet weight (Fig. 4B) and size (Fig. 4C) of the xenograft tumors of the siRNA602 group were significantly decreased compared to those of the NC group. Additionally, the expression of NOTCH1, HEY1, Ki-67, Caspase-3 and Caspase-9 in the xenograft tumors was detected by immunohistochemistry. The expression of NOTCH1 and HEY1 were significantly decreased in the siRNA602 group. The proliferation index (Ki-67) of xenograft tumors of the siRNA-602 group was decreased, while the expression of apoptosis-related proteins (Caspase-3 and Caspase-9) was increased compared with that in the NC group (Fig. 4E, 4F). Collectively, the above results indicate that the inhibition of HEY1 expression in salivary adenoid cystic carcinoma cells can obviously repress tumor formation and growth and induce cell apoptosis *in vivo*.

### HEY1 increases cell migration and invasion *in vitro*

**Figure 5:**
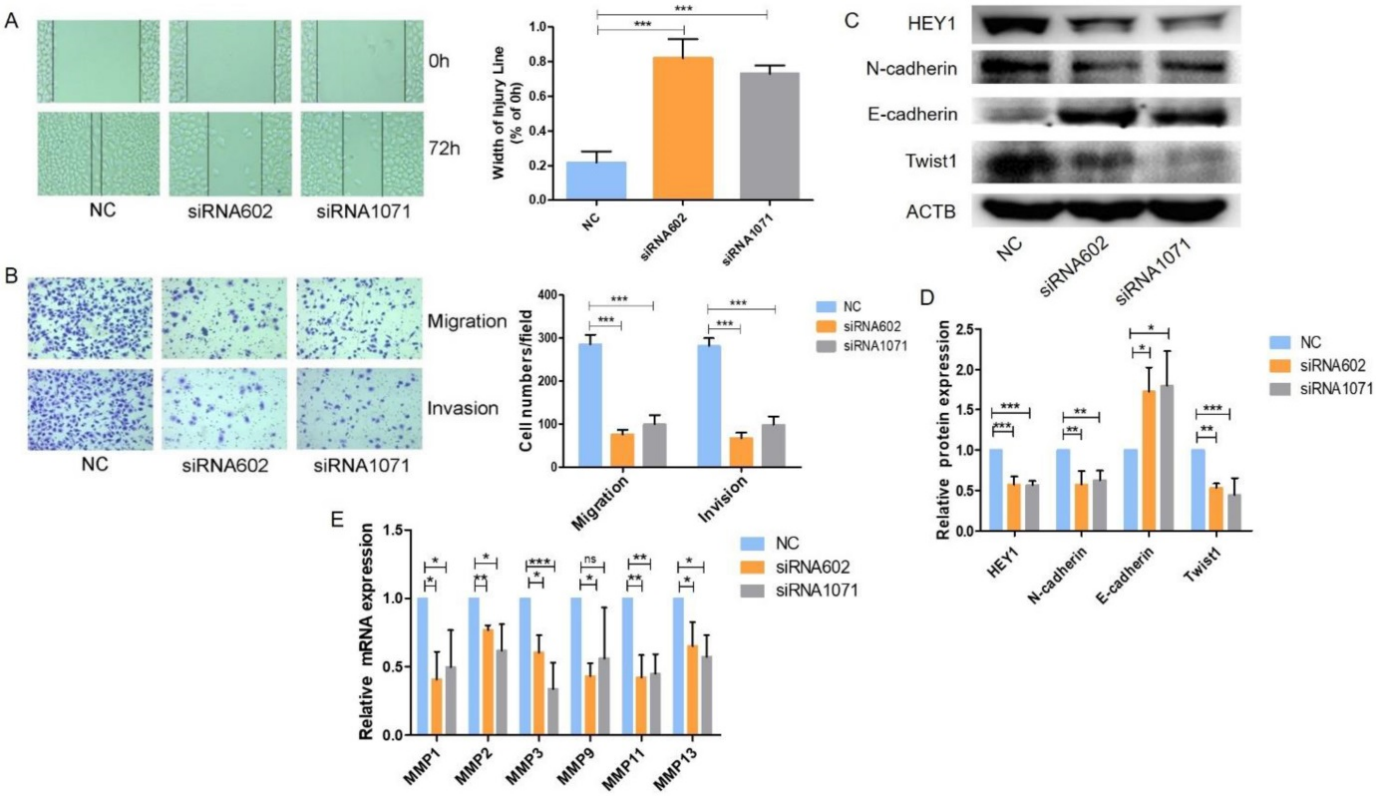
** HEY1 knockdown inhibits SACC cell migration and invasion, and regulates the epithelial-mesenchymal transition and MMP expression in SACC cells.** After inhibited the exression of HEY1, the cell migration and invasion were measured by, wound healing assay (A) and transwell assay coated with or without matrigel(B, P < 0.001 by One-Way ANOVA followed by Tukey's multiple Comparison test), the representative images of transwell chambers coated without (upper panel) or with (lower panel) Matrigel. (C, D) Western blot analysis and the quantification of HEY1, E-cadherin, N-cadherin and Twist1 expression in SACC cells after inhibiting HEY1. (E) The mRNA expression of MMP1, MMP2, MMP3, MMP9, MMP11 and MMP13 in SACC cells was detected by real-time PCR when HEY1 was inhibited. *P < 0.05, ** P < 0.01, and *** P < 0.005.

Next, we examined the migration and invasion of SACC cells with reduced expression of HEY1. As shown in Fig. 5, transfection of the HEY1-specific siRNAs into SACC-LM cells significantly inhibited cell motility and invasion, as indicated by the wound-healing (Fig. 5A) and transwell (Fig. 5B) assays. These findings are concordant with the idea that HEY1 might act as an oncogene in SACC by contributing to the migration and invasion of SACC cells.

### HEY1 regulates the Epithelial-Mesenchymal Transition and MMPs expression in SACC cells

We further explored the underlying molecular mechanisms related to HEY1 and SACC biological properties. It has been reported that HEY1 expression is associated with the epithelial-mesenchymal transition (EMT) 22. Tumor cells need to undergo partial or full epithelial-mesenchymal transition and are transformed into migrating and invasive cells 23. In cancer, the EMT is associated with tumorigenesis, invasion, metastasis, tumor stemness and resistance to therapy 24. Therefore, we measured the expression of EMT-related genes when HEY1 was inhibited in SACC cells (Fig. 5C, 5D). The expression of a mesenchymal marker (N-cadherin) was decreased in the siRNA602 and siRNA1071 groups, while the epithelial marker E-cadherin was significantly increased compared to the NC group. The transcription factor Twist1, which is known to promote tumor metastasis and induce the EMT, was obviously reduced when HEY1 was knocked down. In addition, we also detected the expression of matrix metalloproteinases (MMPs) in SACC cells transfected with different siRNAs (NC, siRNA602 and siRNA1071). The results showed that the knockdown of HEY1 in SACC-LM cells suppressed the expression of MMP1, MMP2, MMP3, MMP9, MMP11 and MMP13 (Fig. 5E). Thus, these results reveal that HEY1 activation drives the EMT and promotes the expression of MMPs in SACC.

### HEY1 and HES1 contribute equally in mediating Notch1 signaling pathway in SACC cells

**Figure 6:**
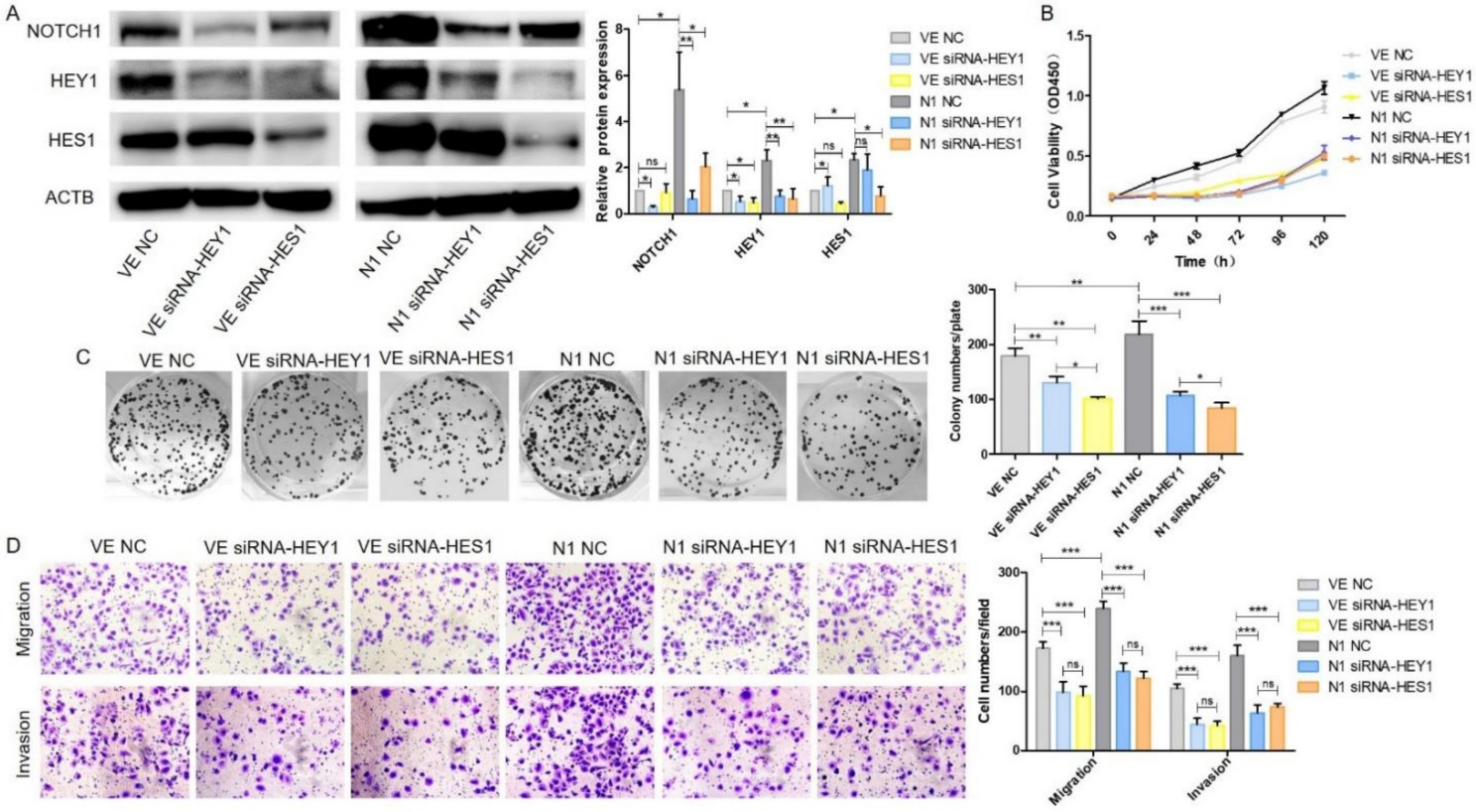
** HEY1 and HES1 contribute equally in mediating NOTCH1 signaling pathway in SACC cells.** After co-transfection with different plasmid DNA and siRNAs at the same time, the protein expression of NOTCH1, HEY1 and HES1 were measured by western blot (A), the cell proliferation was detected by CCK8 assay (B, P < 0.001 by One-Way ANOVA followed by Tukey's multiple Comparison test from 24 h to 120 h) and colony formation assay (C, P<0.05 by One-Way ANOVA followed by Tukey's multiple Comparison test, n=3). The cell migration and invasion were detected by transwell assay coated with (lower panel) or without (upper panel) matrigel (D, 200×, P<0.005 by One-Way ANOVA followed by Tukey's multiple Comparison test ).

We have demonstrated that HEY1 and HES1 16 could promote proliferation, invasion and migration in SACC. They both played an important role in the NOTCH signaling pathway. Therefore, we wondered which would play more important role of NOTCH1 oncogenic function in SACC? As shown in Fig. 6A, after co-transfection of the plasmid DNAs and siRNAs into SACC-LM cells, the expression of NOTCH1, HEY1 and HES1 were significantly elevated in the N1 NC group while compared with the VE NC group. NOTCH1 was obviously inhibited in the condition of HEY1 or HES1 being knockdown in the N1 siRNA-HEY1 group or in the N1 siRNA-HES1 group compared with N1 NC group. Interestingly, the expression of HEY1 was also visibly reduced in the HES1 knockdown group. However, in the HEY1 knockdown group, the same phenomenon was not observed. We speculated that HES1 might regulate the biological behavior of SACC cells by affecting the expression of HEY1 in the NOTCH signaling pathway. The CCK8 (Fig. 6B) showed that the cell proliferation in the N1 NC group was enhanced compared with other groups (P<0.05 at days 2, 3, 4, 5, 6). Compared with the N1 NC group, the cell growth of the N1 siRNA-HEY1 group and the N1 siRNA-HES1 group decreased, but there was no significant difference in the growth inhibition of these two groups (N1 siRNA-HEY1 group vs N1 siRNA-HES1 group, P>0.05 at days 2,3, 5 and 6, P<0.05 at day 4). As expected, the colony formation assays (Fig. 6C) also had the same performance. Moreover, the invasion and migration of SACC cells were boosted in the N1 NC group, and the cell motility and invasion were inhibited in the N1 siRNA-HEY1 group and the N1 siRNA-HES1 group. However, there was no obviously difference in these two groups (Fig. 6D). Taken together, HEY1 and HES1 showed equal importance as a carcinogen in the NOTCH signaling pathway.

## Discussion

The NOTCH signaling cascade is an evolutionarily highly conserved pathway that plays a crucial role in regulating the growth and development of various tissues and maintaining homeostasis. The abnormal activity of this pathway is also closely related to the occurrence and development of several malignant tumors 25. Upon the activation of NOTCH signaling, NOTCH receptors release the signal-transducing NOTCH intracellular domain (NICD). The NICD migrates into the nucleus and combines with the nuclear proteins of the CSL to form the CSL-NICD transcriptional complex and initially target HEY1 directly 26, 27. HEY1 plays crucial roles in the development of various tissues and organs and the occurrence and development of tumors 28-30.

In the classical NOTCH signaling pathway, HEY1 is generally activated by NOTCH receptors. Ilaria Saltarella et al found that the knockdown of NOTCH1/2 in multiple myeloma inhibited the expression of the downstream transcription factor HES1/HEY1, which restrained tumor angiogenesis 30. However, in a study of the association between NOTCH1 and HEY1 in HNSCC, it was shown that HEY1 expression was independent of the activation of NOTCH1, was not associated with either JAG1 or NICD1 expression, and the high expression of HEY1 was associated with a poor prognosis in patients with HNSCC 32. Takahito Fukusumi and colleagues indicated that NOTCH4 specifically activates its downstream target gene HEY1 in HNSCC. Conversely, HEY1 can also regulate the expression of NOTCH4 17. In our results, we demonstrated that NOTCH1 overexpression increased the expression of HEY1 in SACC cells, whereas the knockdown of HEY1 by siRNAs significantly decreased the expression of NOTCH1. Furthermore, the expression of NOTCH1 and HEY1 exhibited clearly positive correlation in adenoid cystic carcinoma tissues. Nonetheless, it is still unknown whether HEY1 regulates NOTCH1 expression through direct regulation or another pathway. These results indicate that the NOTCH1-HEY1 pathway could generate a positive feedback loop instead of one-way regulation in SACC.

Wang et al reported that compared with adjacent tissues, HEY1 expression was significantly elevated in Kaposi's sarcoma tissue, and HEY1 promoted tumor angiogenesis 33. Jung et al. examined the relationship between clinicopathological factors and HEY1 expression in 109 cases of papillary thyroid carcinoma. HEY1 immunoreactivity was positively correlated with lymph node metastasis, recurrence and metastasis 34. However, Ménard M et al showed that HEY1 inhibited tumor progression by regulating tropomyosin receptor kinase C (TrkC) activity in neuroblastoma 35. Through the anta gonistic effects of HEY1 on androgen receptor (AR), NOTCH signaling can possibly antagonize malignant prostate growth 29. HEY1 plays different roles in different tumors. To date, there is no related report on the role of HEY1 in SACC. Our immunohistochemistry results showed that the expression of HEY1 in salivary adenoid cystic carcinoma was significantly higher than that in adjacent tissues. We speculated that HEY1 might promote the occurrence and development of SACC. Brian C. Belyea and colleagues found that abnormal NOTCH-HEY1 signaling promotes tumor progression by blocking cell differentiation and promoting cell proliferation in embryonic subtypes of rhabdomyosarcoma 36. Thus, we next examined HEY1 functions in SACC and showed that HEY1 enhanced cell proliferation *in vitro* and tumorigenicity *in vivo*. In addition, the results of our apoptosis analysis revealed that HEY1 inhibited cell apoptosis. These results suggest that HEY1 promotes the growth of SACC by regulating cell proliferation and apoptosis.

Previous findings have mainly focused on the relationship between HEY1 and the development of the cardiovascular system. Luis Luna-Zurita et al reported that HEY1 affected endocardial formation by regulating the myocardial EMT and the expression of BMP2 in nonchamber myocardium 37. In cancer, HEY1 has been reported to be essential for the transforming growth factor-β-dependent EMT, which is frequently observed in advanced carcinogenesis and is related to several cancer-related pathways 28. Tumor cells transition between epithelial and mesenchymal states in a highly plastic and dynamic manner 38. The epithelial-mesenchymal transition (EMT) is a reversible process that promotes cell invasion in which cells lose their epithelial characteristics and acquire mesenchymal characteristics, altering the adhesion molecules on the cell surface and allowing them to migrate more efficiently and invade potential mesenchyme 39-41. In a study of HNSCC, HEY1 promoted cell invasion and migration by inducing the epithelial-mesenchymal transition and enhanced stemness 32. In our research, we also focused on the relationship between HEY1 and the EMT. The results showed that N-cadherin and Twist1 were significantly decreased, while E-cadherin was obviously increased following the inhibition of HEY1 in SACC cells. The EMT is essential for cancer invasion and metastasis 42. Our *in vitro* experiments showed that HEY1 promoted the migration and invasion of SACC cells. We believe that HEY1 promotes cell invasion and metastasis by regulating the epithelial-mesenchymal transition in SACC.

The NOTCH pathway has been reported to modulate the homeostasis of cancer stem cell-like cells (CSCs) in colorectal cancer 43. Miranda Brun et al found that HEY1 was associated with increased proliferation and stem cell characteristics in GBM cells 44. The EMT is closely related to CSCs and tumor resistance 45. The EMT is thought to be induced in a subset of metastatic cancer stem cells (MCSCs), giving the tumor the ability to metastasize throughout the body 46. After inhibiting the expression of HEY1 in SACC, we found that the volume and number of tumor cell spheroids were significantly reduced. The EMT-induced genes and CSC markers Snail1, Twist1 and Fibronectin 1 were decreased after HEY1 expression was downregulated. In addition, we also examined the expression of OCT4 and ALDH1, known CSC markers, which was also downregulated. Aldehyde dehydrogenase 1 (ALDH1) is a cancer stem cell-like cell (CSC) marker in human cancer that is involved in the regulation of NOTCH1 signaling and regulates the formation of ovarian cancer tumor spheres 38, 47. In HNSCC, OCT4 is considered a key regulator of CSCs, which targets CSCs and may be of potential value in the treatment of HNSCC 48.

Furthermore, in cancer, abnormally hydrolyzed matrix metalloproteinases cause uncontrolled tumor growth, tissue remodeling, inflammation, tissue invasion and metastasis 49. Pope JL et al found that claudin-1 regulates Notch signaling through the regulation of MMP-9 and p-ERK signaling to regulate intestinal epithelial homeostasis 50. Qi Zhang et al reported that MMP2, HEY1 and HES1 together regulate the migration of adipose-derived stem cells and chondrocytes in a coculture system 51. In tumor studies, MMPs induced tumor invasion and distant metastasis in a variety of tumors, such as breast cancer 52, osteosarcoma 53, endometrial cancer 54, and esophageal squamous cell carcinoma 55. Therefore, we also studied the correlation between HEY1 and MMPs. Real-time PCR analysis revealed that HEY1 positively regulated the expression of MMP1, MMP2, MMP3, MMP9, MMP11 and MMP13. However, it is still unknown how HEY1 affects the expression of MMPs. Zhang et al found that unbalanced expression of MMP/TIMP axis genes in tumors was corrected with a simple defined factor-mediated reprogramming for nuclear remodeling to change the morphology of tumor cells and to inhibit the migration and invasion of tumor cells 56. MMP is widely used as a cancer biomarker and therapeutic target. Recently, MMP-based nanomaterials and carriers have emerged in the tumor-targeted delivery of drugs and as imaging agents at the tissue, cellular, and intracellular levels 57. These results will also provide a new direction for the invasion and metastasis of SACC based on HEY1 and MMPs.

In summary, we demonstrate that the NOTCH1-HEY1 pathway is specifically upregulated in SACC and promotes the EMT and MMP expression. HEY1 regulates cell proliferation, spheroid formation, invasion, metastasis and apoptosis inhibition *in vitro* and *in vivo*. Therefore, this finding provides new insight into the role of the NOTCH signaling pathway in SACC carcinogenesis.

## Figures and Tables

**Table 1:** The sequences of the siRNAs used in the current study

Name	Sequence		
siRNA-HEY1-602	5'-CGCGUUAUCUGAGCAUCAUTT-3'		5'-AUGAUGCUCAGAUAACGCGTT-3'
siRNA-HEY1-1071	5'-GGCAAGCCCUAUAGACCUUTT-3'		5'-AAGGUCUAUAGGGCUUGCCTT-3'
siRNA-HES1-670	5'-CCAACUGCAUGACCCAGAUTT-3'		5'-AUCUGGGUCAUGCAGUUGGTT-3'
NC	5'-UUCUCCGAACGUGUCACGUTT-3'		5'-ACGUGACACGUUCGGAGAATT-3'
			

**Table 2:** The primers for real-time PCR and semiquantitative RT-PCR used in the current study

Gene	Forward	Reverse
ACTB	5'-CCTGGCACCCAGCACAAT-3'	5'-GGGCCGGACTCGTCATACT-3'
HEY1	5'-CGAGGTGGAGAAGGAGAGTG-3'	5'-CTGGGTACCAGCCTTCTCAG-3'
NOTCH1	5'-GGAAGTTGAACGAGCATAGTCC-3'	5'-GCATGATGCCTACATTTCAAGA-3'
TNNT3	5'-ATTCGTGCAGAGAAGGAGAGGG-3'	5'-TCTTGCCTCTCTTCTGGTCAGC-3'
IFI6	5'-CTACCTGCTGCTCTTCACTTGC-3'	5'-TCCTCCGACGGCCATGAAG-3'
RELB	5'-CGTGCATGCTTCGGTCTGG-3'	5'-GCCGTTCTCCTTGATGTACTCG-3'
KRT-17	5'-AGGACAATTGAGGAGCTGCAGA-3'	5'-ATTGATGTCGGCCTCCACACT-3'
MMP7	5'-CATGAGTGAGCTACAGTGGGA-3'	5'-CTATGACGCGGGAGTTTAACAT-3'
TPD52	5'-CTGTTGGCTCAGTCATCACCAA-3'	5'-CTTTTCTGGAAGAGGCTCCGTG-3'
ABHD3	5'-TACCCTTCTGCTCCTTTCCTGG-3'	5'-TCCAATGACTCTGAGCAAGCGA-3'
Snail1	5'-GCCTTCAACTGCAAATACTGC-3'	5'-CTTCTTGACATCTGAGTGGGTC-3'
Twist1	5'-CCATGTCCGCGTCCCACTA-3'	5'-CCCACGCCCTGTTTCTTTGAAT-3'
Fibronectin 1	5'-GCCGAGGTTTTAACTGCGAGAG-3'	5'-CGATGCAGGTACAGTCCCAGAT-3'
MMP1	5'-GGGGAGATCATCGGGACAACTC-3'	5'-AGAATGGCCGAGTTCATGAGCT-3'
MMP2	5'-CAAGGACCGGTTCATTTGGC-3'	5'-GGCCTCGTATACCGCATCAA-3'
MMP3	5'-TCAGAACCTTTCCTGGCATCCC-3'	5'-CAGCCTGGAGAATGTGAGTGGA-3'
MMP9	5'-GCCCGACCCGAGCTGACTC-3'	5'-TTCAGGGCGAGGACCATAGAGG-3'
MMP11	5'-CCGTGCTGACATCATGATCGAC-3'	5'-CAAATTCATGGGCTGCCACCTG-3'
MMP13	5'-TTTCAACGGACCCATACAGTTTG-3'	5'-CATGACGCGAACAATACGGTTA-3'
